# Toxicity and Disease-Related Outcomes after Radiotherapy for Head and Neck Cancer in Human Immunodeficiency Virus-Positive Patients

**DOI:** 10.3389/fonc.2014.00316

**Published:** 2014-11-10

**Authors:** David J. Grew, Benjamin T. Cooper, Susanna Nguy, Jason Halperin, Nicholas J. Sanfilippo

**Affiliations:** ^1^Department of Radiation Oncology, New York University School of Medicine, New York, NY, USA; ^2^Department of Infectious Disease, New York University School of Medicine, New York, NY, USA

**Keywords:** human immunodeficiency virus, head and neck cancer, radiotherapy, squamous cell carcinoma, oropharynx

## Abstract

**Background:** Human immunodeficiency virus (HIV) seropositivity may be associated with higher risk of local recurrence and poor survival in multiple malignancies. However, long-term disease control in HIV-positive patients with head and neck cancer (HNC) is not well described. The purpose of this study is to review the disease-related outcomes of HIV-positive patients who underwent radiotherapy (RT) or chemoradiotherapy (CRT) at our institution.

**Methods:** We retrospectively reviewed 24 HIV-positive patients who underwent RT for HNC between 2004 and 2013. Patient characteristics, treatment details, and outcomes were collected. Overall survival (OS) and local recurrence-free survival (LRFS) were investigated. Kaplan–Meier estimated survival was calculated.

**Results:** Median follow-up was 21 months. All patients were treated with curative intent. Eighty-three percent had stage III–IV. Primary sites of disease included oropharynx (*n* = 12), larynx (*n* = 6), oral cavity (*n* = 2), unknown primary (*n* = 2), nasal cavity (*n* = 1), and paranasal sinuses (*n* = 1). Four patients (17%) had definitive RT alone and nine had definitive CRT (38%; eight cisplatin and one cetuximab). Eleven (46%) were treated in the adjuvant setting after surgical resection; six with RT alone and five with concurrent cisplatin. Eight patients had acute Grade 3 toxicity with no acute Grade 4 or 5 toxicities. Fifteen patients (63%) were alive and disease-free. Two- and 5-year OS was 67 and 59%, respectively. LRFS at 2-years was 82%. Median OS was 83 months.

**Conclusion:** In this cohort, HIV-positive patients treated aggressively with curative intent had excellent OS and local control following RT or CRT for HNC compared to historical controls. Treatment was relatively well tolerated. This group of patients should be managed aggressively with intent to cure.

## Introduction

In the highly active anti-retroviral therapy (HAART) era, human immunodeficiency virus (HIV)-positive patients are living longer and are at higher risk for developing non-AIDS-defining malignancies (1–3). The incidence of head and neck cancer (HNC) in particular, has increased markedly since the widespread use of HAART (3–6). Despite becoming a more common problem, the optimal management of HNC in HIV-positive patients is unknown because of a paucity of data.

In HIV-positive patients with anal squamous cell carcinoma (SCC), some have argued against aggressive therapy in patients with advanced disease, citing concerns regarding increased toxicity after chemoradiation (CRT) (7, 8). The combined modality treatment of HNC with concurrent CRT in both the definitive and adjuvant setting results in improved disease outcomes at the cost of added toxicity (9–12). However, the magnitude of increased toxicity in HIV-positive patients is not well described. Some reports suggest that the acute toxicity of radiotherapy (RT) or CRT in HIV-positive HNC patients is comparable to that of HIV-negative patients (13–15). However, data from Mourad et al. suggest that RT is less tolerated among HIV-positive patients (16), possibly narrowing the therapeutic window in this fragile patient population.

Data on disease-related outcomes of HIV-positive patients with HNC are lacking. However, this information is essential for oncologists to effectively weigh the certainty of treatment toxicity against the potential benefit of aggressive treatment when making management decisions in this patient population. Furthermore, a better understanding of disease control and survival rates aids in counseling patients on reasonable treatment expectations. Here, we review our single institution experience treating HNC in HIV-positive patients over a 10-year period and describe clinical outcomes. Secondary objectives include reporting acute toxicity, patterns of failure, and other factors, which may affect prognosis in HIV-positive HNC patients.

## Materials and Methods

### Patients

Approval of the Institutional Review Board was obtained for this retrospective study. All patients who received radiation therapy to the head and neck in our department between 2004 and 2014 were reviewed. Our radiation oncology department services patients from both a hospital that mainly accepts patients with private insurance and a public, community-based hospital, which is obligated to treat prisoners, underinsured, and uninsured patients. Patients from both institutions were included in this analysis. HIV-positive patients with cancer of the aerodigestive tract were identified. Two of 13 patients from our previous report on acute toxicity were excluded (squamous cell cancer of the skin and parotid hyperplasia) from this analysis (13). The remaining 11 patients and 13 additional patients were included and evaluated for long-term disease-related outcomes. Patient and treatment-related data that were examined included age, gender, histologic diagnosis, primary disease site, stage of disease, human papillomavirus (HPV) status, HAART therapy, RT technique [three-dimensional conformal radiotherapy (3D-CRT) or intensity modulated radiotherapy (IMRT)], total RT dose, duration of RT, and the presence of concurrent chemotherapy. HIV-related information examined included pre-treatment CD4+ lymphocyte count and viral load. Patient toxicity data were manually garnered from the radiation and medical oncology treatment charts. All initial consultation, weekly radiation on-treatment, medical oncology on-treatment, and follow-up notes were examined. The electronic medical record of the referring hospital system was systematically queried in addition to the paper charts for every patient.

### Treatment and follow-up

All treatments were delivered with megavoltage photon or electron beams. In patients receiving concurrent chemotherapy, cisplatin 100 mg/m^2^ was given every 3 weeks in all but one patient, who received cetuximab 400 mg/m^2^ loading dose followed by 250 mg/m^2^ weekly for the duration of radiation therapy. Patients were evaluated by a radiation oncologist on a weekly basis during RT, then monthly in multidisciplinary clinic with an otolaryngologist, radiation oncologist, and medical oncologist following RT completion for 3 months, then every 3 months. Acute and late toxicity was assessed as well as any treatment-related complications. Treatment outcomes that were examined included presence of local recurrence, distant metastasis, and death. HIV specific follow-up was at the discretion of the treating infectious disease specialist.

### Statistics

Chi-square test was used to compare frequencies and *t*-test was used to compare means. Overall survival (OS) was defined as the duration from the start of RT to most recent follow-up or death. Local recurrence-free survival (LRFS) was defined as time from start of RT to most recent follow-up, tumor recurrence at the primary site or death. Survival estimates were calculated according to the Kaplan–Meier method. All statistical analyses were performed using SPSS version 20 (SPSS Inc., Chicago, IL, USA).

## Results

### Patient characteristics

Twenty-four HIV-positive patients underwent RT for HNC of the aerodigestive tract. Median age was 53 (range, 32–67); 4 patients were women, 20 were men. Twenty-three patients had SCC, one patient had sarcomatoid SCC (Table 1). Primary disease sites included oropharynx (*n* = 12), larynx (*n* = 6), oral cavity (*n* = 2), unknown primary (*n* = 2), nasal cavity (*n* = 1), and paranasal sinuses (*n* = 1). Three patients (13%) had stage II disease, 5 (21%) had stage III, 14 (58%) had IVA, and 2 (8%) had IVB. Sixteen patients reported smoking ever; four patients reported ≥10 pack-years smoking history.

**Table 1:** **Patient and disease characteristics**.

Characteristic	Total patients (%)
Gender
Male	20 (83)
Female	4 (17)
Histology
SCC	23 (96)
Sarcomatoid SCC	1 (4)
Primary site
Oropharynx	12 (50)
Larynx	6 (25)
Oral cavity	2 (8)
Unknown primary	2 (8)
Nasal cavity	1 (4)
Paranasal sinuses	1 (4)
T Stage
Tx	1 (4)
T0	2 (8)
T1	3 (13)
T2	7 (29)
T3	5 (21)
T4	6 (25)
N Stage
N0	8 (33)
N1	4 (17)
N2	10 (42)
N3	2 (8)
AJCC seventh edition stage group
II	3 (13)
III	5 (21)
IVA	14 (58)
IVB	2 (8)

*SCC, squamous cell carcinoma; AJCC, American Joint Committee on Cancer*.

### Survival

**Figure 1:**
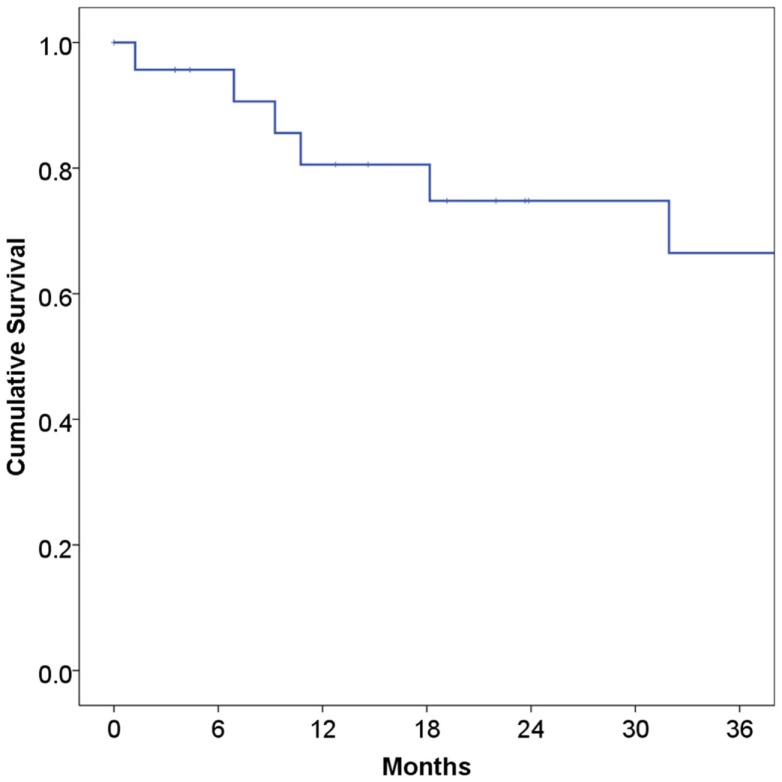
**Overall survival among human immunodeficiency virus-positive patients undergoing radiotherapy for head and neck cancer**.

**Figure 2:**
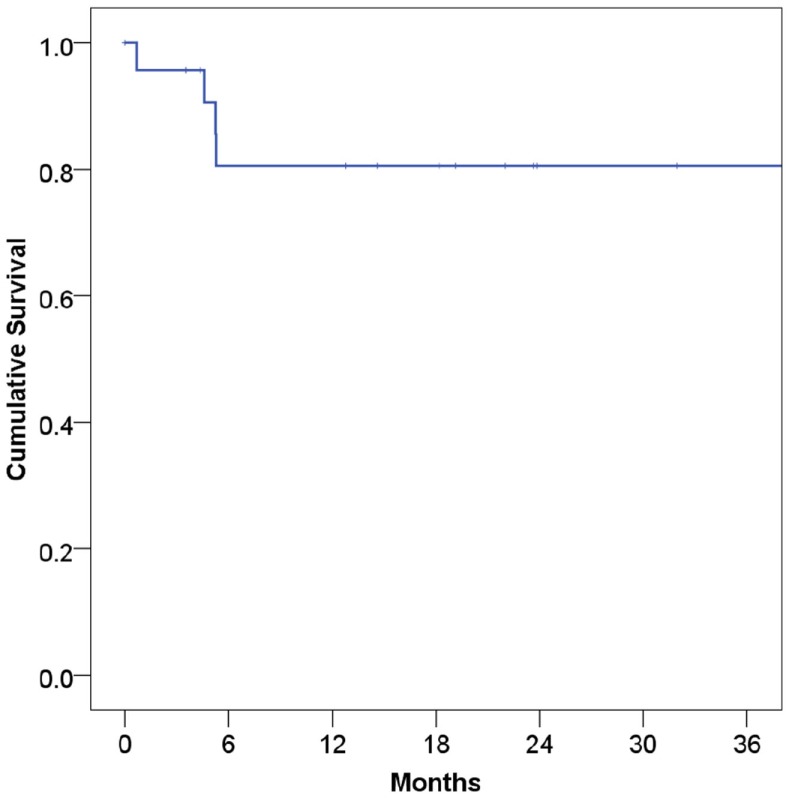
**Local recurrence-free survival among human immunodeficiency virus-positive patients undergoing radiotherapy for head and neck cancer**.

At a median follow-up of 21 months, 16 patients (67%) were alive and 15 (63%) were alive and free of disease. Projected 2 and 5-year OS was 67 and 59%, respectively (Figure 1). Median OS was 83 months (95% CI 9.4–156 months). Causes of death include widespread metastases (*n* = 4), progression of primary (*n* = 2), sepsis during chemotherapy for second primary malignancy (lung) (*n* = 1), and unknown (*n* = 1). Of note, no patients died of complications from acquired immunodeficiency. Projected 2-year LRFS was 82% (Figure 2), median LRFS was not reached. Of the patients who experienced local failure, primary sites of disease included base of tongue (*n* = 3) and retromolar trigone (*n* = 1).

### HIV-related parameters

Data on HAART were available for 22 patients, 19 (86%) of which were on HAART at the time of consultation. All 19 patients on HAART continued therapy concurrently with RT. Twelve patients were taking protease inhibitors concurrently with RT. CD4+ lymphocyte counts were available for 12 patients. Median CD4+ cell count was 261 cells/μL (range 71–858 cells/μL). HIV viral load was available for 12 patients. Ten patients had an undetectable viral load (<50 copies/mL), one had a viral load 687 copies/mL, and one patient not on HAART had viral load 46,600 copies/mL. HPV status was available for three patients, two of whom were HPV-positive. There was no significant difference in rates of death, local recurrence, or distance metastases among patients taking HAART medications. There was also no significant difference in the rate of death, local recurrence, or metastases among patients taking protease inhibitors.

### Radiotherapy details

Radiotherapy was delivered by 3D-CRT for 15 patients (63%) and IMRT for 9 (37%). Four patients (17%) had definitive RT alone and nine CRT (38%; eight cisplatin and one cetuximab). Eleven (46%) were treated in the adjuvant setting after surgical resection, six with RT alone and five with concurrent cisplatin. Median RT dose for patients undergoing definitive treatment was 70 Gy (range 35–70.4 Gy) and median RT dose for patients undergoing post-operative treatment was 66 Gy (range 54–70 Gy). Three patients (13%) terminated treatment prematurely. One patient had progression of disease and treatment was terminated at 35 Gy. The patient expired 12 days later. Two patients stopped coming in for treatment mid-course and were lost to follow-up despite multiple phone calls and sending a certified letter. All patients were treated with conventional fractionation daily, 5 days a week. Two patients received 1.8 Gy per fraction, all others received 2 Gy per fraction. Median RT course duration was 53 days (range 21–71 days).

### Hematologic details

The median pre-treatment white blood cell count, absolute neutrophil count (ANC), hemoglobin level, and platelets were 6,100 cells/μL, 3,700 cells/μL, 11.1 g/dL, and 240,000 cells/μL, respectively. The median nadir of the white blood cell count, ANC, hemoglobin level, and platelets were 2,200 cells/μL, 1,200 cells/μL, 8.9 g/dL, and 145,000 cells/μL, respectively. One patient receiving chemotherapy and one patient treated definitively with RT alone required hospitalization for neutropenic fever. The patient being treated with RT alone had local disease progression and eventually died of sepsis related to the progressive disease. This corresponded to an 8% rate of admission for neutropenic fever in the entire cohort. Eight of 24 patients became neutropenic (ANC < 1,000 cells/μL) at some point during their treatment, most of whom were treated with granulocyte colony-stimulating factor support. Interestingly, three of the patients who became neutropenic were not receiving chemotherapy.

### Toxicity

Grade 0, 1, and 2 toxicities are displayed in Table 2. There were no Grade 4 or 5 acute toxicities in this series. Eight patients (33%) experienced Grade 3 toxicity: three had Grade 3 dermatitis, four had Grade 3 mucositis, and one had Grade 3 esophagitis (Table 2). One patient with T4aN2b base of tongue cancer had laryngeal edema necessitating an extended treatment break, resulting in a treatment time of 71 days total. Three patients had oropharyngeal thrush during RT, which resolved with topical antifungal treatment. Median weight loss in the entire cohort was 5.9 kg (range 2.3–13.6 kg). Median weight loss in the patients treated with or without concurrent chemotherapy were 7.5 and 5.5 kg, respectively. This corresponded to a weight loss as a percentage of total body weight 11.2% (range −8 to 30%) in patients receiving concurrent chemotherapy compared to 7.4% (range 3–19%) in patients without combined therapy. The only patient that gained weight during treatment (−8% weight loss) was because of PEG tube placement after a difficult post-operative course.

**Table 2:** **Acute toxicity: RTOG common toxicity criteria and total numbers of patients affected**.

Acute toxicity	Grade 0	Grade 1	Grade 2	Grade 3	Grade 4
Dermatitis	1	7	5	3	0
Mucositis	2	3	8	4	0
Fatigue	16	0	1	0	0
Esophagitis	9	3	4	1	0
Nausea	14	1	1	0	0
Anorexia	14	2	1	0	0
Xerostomia	3	7	5	0	0

*RTOG, radiation therapy oncology group*.

There were no significant increases in the frequency of Grade 3 toxicity among patients taking HAART. Severe late RT complications were rare. One patient had wound dehiscence after adjuvant RT for T1N2b tonsillar cancer. He did not receive concurrent chemotherapy and was not on HAART. One patient developed a tracheoesophageal fistula after adjuvant CRT to for T3N2c supraglottic larynx cancer. One patient T1N2a supraglottic larynx cancer treated with definitive CRT to 70 Gy was PEG-dependent until his death in 2010.

## Discussion

In our series, HIV-positive patients treated with definitive or adjuvant RT or CRT for HNC had 67% OS and 82% local control at a median follow-up of 2 years, comparing favorably with historical data. In addition, treatment was well-tolerated with limited acute toxicity and rare late complications. Further, while highly curable, HNC in HIV-positive patients remains potentially life-threatening. In our series, three-quarters of all patient deaths were a result of local or distant progression of disease. These findings provide further evidence to support a curative approach to HIV-positive patients with HNC.

Historically, advanced HNC has been a difficult disease to manage, with morbid treatment and poor long-term disease-related outcomes. The expected 2-year OS and local control after definitive CRT for stage III–IV HNC in immunocompetent patients range from 40 to 75% and 60 to 80%, respectively (11, 17, 18). Results after surgical resection and adjuvant CRT are similar, with approximately 65–75% survival and 80–85% local control at 2-years (9, 10). Klein et al. published the first report on disease outcomes among HIV-positive patients (14). While their study is limited by small sample size (*n* = 12), they report excellent results with 78% OS and 92% local control at 3-years. More recently, Mourad et al. reported 69% locoregional control and 55% OS at 4 years among 73 HIV-positive patients treated with definitive RT (19).

There are several possible explanations for these favorable results. First, we treated all patients aggressively, with multimodality treatment where appropriate. Our treatment strategy is not modified based on HIV status. While each patient is approached individually, our general practice is CRT for definitive treatment of stage III/IV HNC and adjuvant CRT for high-risk pathologic features including positive margins and extracapsular extension. These strategies were employed in the majority of patients on this study.

The epidemiology of HNC is changing, and favorable outcomes in our series may be a reflection of this. In recent years, the incidence of HPV-unrelated HNC has declined while HPV-associated HNC is on the rise (20). HPV-positive HNC patients have a significantly better prognosis than HPV-negative patients (21) and high rates of HPV-positivity in our cohort may contribute to the favorable results we observed. Unfortunately, because most patients in this series were treated before HPV testing became institutional policy, we were only able to obtain HPV status from pathologic specimens of three patients. Also, to complicate matters, both referring hospital’s pathology departments were submerged in Fall of 2012 due to a weather related catastrophe, making retrospective HPV analysis of possible stored specimen impossible. There are, however, other characteristics that suggest that a high-HPV-positivity rate in this cohort including younger age at diagnosis (median 53 years), high proportion of oropharyngeal primary site (50%), and lack of heavy smoking history (17% patients had >10 pack-years).

It is possible that the addition of protease inhibitors concurrent with RT contributed to improved outcomes. Protease inhibitors block the Akt signaling pathway and result in synergistic tumor killing when given concurrently with RT (22). In our series, the administration of concurrent protease inhibitor with RT did not result in either worse toxicity or improved disease-related outcomes. However, this analysis is limited by small patient numbers, making it difficult to draw meaningful conclusions based on these data. The impact of protease inhibitors on clinical outcomes requires further exploration as it may have important implications for the widespread application of this therapy for tumors of many disease sites (23).

Acute toxicity in this cohort compares favorably with historical controls. RT alone typically results in <25% Grade 3 or 4 mucositis in immunocompetent patients. The addition of concurrent chemotherapy increases the risk of Grade 3–4 mucositis to 32–84% (24). Here, we observed 33% had any Grade 3 acute toxicity and 17% had Grade 3 mucositis. We did not report rates of late toxicity because follow-up notes did not consistently classify toxicity by RTOG Grade. We determined that retrospectively converting narrative descriptions of late effects from notes to RTOG Grade was an extrapolation that introduced a high degree of uncertainty and bias to these data.

Looking forward, HIV-positive HNC patients may be excellent candidates for molecular targeted therapy. The ongoing RTOG 1016 study randomizes p16-positive HNC patients with clinical stage T1-2, N2a-3, or T3-4, any N to concurrent accelerated RT with either cisplatin or cetuximab. This head to head comparison will determine whether cetuximab results in equivalent survival as compared to cisplatin. The results of this trial are highly anticipated as they may usher in a new era in which chemotherapy is replaced by molecular targeted agents in HPV-positive HNC. This is especially relevant for HIV-positive HNC patients for whom epidemiologic data suggest high rates of HPV-related disease.

The strengths of this study are that it is a single institution experience over 10 years entirely in the HAART era with a consistent curative treatment approach. All patients were treated by the same two radiation oncologists. Limitations include retrospective analysis, which subjects this study to the typical biases, including selection bias. Additionally, as a retrospective study, it relies on the accuracy of follow-up clinic notes and imaging study reports for tabulation of toxicity and the classification of clinical endpoints. However, given the relative rarity of HNC in HIV-positive patients, prospective studies may not be feasible. Additionally, while this represents one of the largest series describing disease-related outcomes in this setting, patient numbers are small.

Finally, despite including 24 patients treated over the course of 10 years, median follow-up is only 21 months. This discordance between duration of the study period and median follow-up is a limitation of the study. A major contributor to the lack of follow-up is a large proportion of patients being referred from a hospital that serves uninsured patients of likely lower socioeconomic backgrounds who are less likely to complete routine follow-up as scheduled. Furthermore, lower rates of retention in the medical care system for HIV-positive patients have been documented and are associated with a lack of medical insurance, lower socioeconomic status, and competing psychosocial needs (25). Loss to follow-up for HIV specialty care is directly associated with increased mortality including death from malignancies (26). Those retained in the care of an infectious disease specialist have frequent follow-up with many health-care practitioners to manage HAART therapy and their other medical co-morbidities. This can lead to the misperception that sub-specialty follow-up is not required, which has been recently shown for missed gynecologic referrals in the setting of abnormal pap tests (27). This study highlights the challenge of establishing reliable follow-up with medical oncology, radiation oncology, and otolaryngology in this patient population, for whom other social issues may interfere with and take precedence over cancer surveillance.

## Conclusion

In this cohort of HIV-positive patients with HNC, RT and CRT were well-tolerated and resulted in survival and disease control rates that compare favorably with historical controls. This group of patients should be managed aggressively with intention to cure. Despite excellent OS, most patient deaths that did occur were a result of disease progression. Premature de-escalation of therapy in this population may result in excess disease-related deaths.

## Conflict of Interest Statement

The authors declare that the research was conducted in the absence of any commercial or financial relationships that could be construed as a potential conflict of interest.
